# Pan-Genome Analysis of Oral Bacterial Pathogens to Predict a Potential Novel Multi-Epitopes Vaccine Candidate

**DOI:** 10.3390/ijerph19148408

**Published:** 2022-07-09

**Authors:** Tehniyat Rida, Sajjad Ahmad, Asad Ullah, Saba Ismail, Muhammad Tahir ul Qamar, Zobia Afsheen, Muhammad Khurram, Muhammad Saqib Ishaq, Ali G. Alkhathami, Eid A. Alatawi, Faris Alrumaihi, Khaled S. Allemailem

**Affiliations:** 1 Department of Health and Biological Sciences, Abasyn University, Peshawar 25000, Pakistan; tehniyatrida88@gmail.com (T.R.); asadullahaup@gmail.com (A.U.); zobia.afsheen@abasyn.edu.pk (Z.A.); saqib.ishaq@abasyn.edu.pk (M.S.I.); 2 Department of Biological Sciences, National University of Medical Sciences, Rawalpindi 46000, Pakistan; sabaismail7@gmail.com; 3 Department of Bioinformatics and Biotechnology, Government College University, Faisalabad 38000, Pakistan; tahirulqamar@gcuf.edu.pk; 4 Department of Pharmacy, Abasyn University, Peshawar 25000, Pakistan; muhammad.khurram@abasyn.edu.pk; 5 Department of Clinical Laboratory Sciences, College of Applied Medical Sciences, King Khalid University, Abha 61481, Saudi Arabia; agaithan@kku.edu.sa; 6 Department of Medical Laboratory Technology, Faculty of Applied Medical Sciences, University of Tabuk, Tabuk 71491, Saudi Arabia; eid.alatawi@ut.edu.sa; 7 Department of Medical Laboratories, College of Applied Medical Sciences, Qassim University, Buraydah 51452, Saudi Arabia; f_alrumaihi@qu.edu.sa

**Keywords:** *Porphyromonas gingivalis*, pan-genomics, immunoinformatics, epitope vaccine, molecular dynamics simulations

## Abstract

*Porphyromonas gingivalis* is a Gram-negative anaerobic bacterium, mainly present in the oral cavity and causes periodontal infections. Currently, no licensed vaccine is available against *P. gingivalis* and other oral bacterial pathogens. To develop a vaccine against *P. gingivalis*, herein, we applied a bacterial pan-genome analysis (BPGA) on the bacterial genomes that retrieved a total number of 4908 core proteins, which were further utilized for the identification of good vaccine candidates. After several vaccine candidacy analyses, three proteins, namely lytic transglycosylase domain-containing protein, FKBP-type peptidyl-propyl cis-trans isomerase and superoxide dismutase, were shortlisted for epitopes prediction. In the epitopes prediction phase, different types of B and T-cell epitopes were predicted and only those with an antigenic, immunogenic, non-allergenic, and non-toxic profile were selected. Moreover, all the predicted epitopes were joined with each other to make a multi-epitopes vaccine construct, which was linked further to the cholera toxin B-subunit to enhance the antigenicity of the vaccine. For downward analysis, a three dimensional structure of the designed vaccine was modeled. The modeled structure was checked for binding potency with major histocompatibility complex I (MHC-I), major histocompatibility complex II (MHC-II), and Toll-like receptor 4 (TLR-4) immune cell receptors which revealed that the designed vaccine performed proper binding with respect to immune cell receptors. Additionally, the binding efficacy of the vaccine was validated through a molecular dynamic simulation that interpreted strong intermolecular vaccine–receptor binding and confirmed the exposed situation of vaccine epitopes to the host immune system. In conclusion, the study suggested that the model vaccine construct has the potency to generate protective host immune responses and that it might be a good vaccine candidate for experimental in vivo and in vitro studies.

## 1. Introduction

Antibiotics are drugs used to hinder the growth of bacteria or kill it. The indiscriminate use of antibiotics pushes bacteria to evolve novel antibiotic resistance mechanisms resulting in economic losses and high mortality [1,2]. The increasing resistance of microbes towards antibiotics is the greatest challenge for mankind. The antibiotic resistance causes 33,000 deaths each year. In Thailand, antibiotic resistance causes almost 38,000 deaths and in the US, the death rate is about 23,000 deaths [3]. The total estimated deaths caused by antimicrobial resistance per year is 70,000 [4,5]. The problem of resistance arises due to the overuse and inappropriate use of antibiotics [6]. To combat antibiotic-resistant bacterial pathogens, the development of antibodies (immunotherapeutic) that specifically target infectious pathogens is an attractive technique [7]. On the other hand, antibiotic-resistant pathogen transmission can be controlled by developing vaccines to provide acquired active immunity to the host [8]. As there is limited availability of vaccines for treating health-associated infections (HAIs), there is an urgent need to accelerate and open new avenues for advancing vaccine development [9]. A safe, specific, and potent vaccine thus can be tagged as the “the need of the hour” [7,10,11]. For combating antibiotic resistance, methods that nurture the immune system of humans by immunological and immunotherapeutic mediation [12] are an attractive and effective approach. The therapeutic solutions which are used for the treatment of pathogens are limited because these pathogens evade innate defenses [13]. For combating a disease, both drugs and vaccines are used, and resistance develops against both. However, resistance to drugs by bacterial pathogens is quick while resistance to vaccines is rare [14]. The reason behind the resistance of drugs is its therapeutic nature as it is given after infection while the vaccine is prophylactic as it is given before infection as a preventive measure. Additionally, another reason for drug resistance is that they are used against very few targets while vaccines are used against various targets [15].

*Porphyromonas gingivalis* is an oral bacterial pathogen and is responsible for chronic periodontitis [16]. *P. gingivalis*, a Gram-negative, black-pigmented anaerobic rod residing in subgingival biofilms, is a causative agent of periodontal diseases along with other oral microorganisms. This bacteria has been additionally thought to cause coronary illness, stroke, and diabetes mellitus [16,17,18]. Periodontal disease is initiated by oral bacteria perturbing epithelial cells, thereby triggering innate, inflammatory, and adaptive immune responses. More than 500 bacterial species interact with human tissues in the human oral cavity. Of these species, *Treponema denticola*, *Aggregatibacter actinomycetemcomitans*, *P. gingiyalis*, *Tannerella forsythia*, *Campylobacter rectus* and *Fusobacterium nucleatum* are associated with periodontitis [19]. No vaccine is currently licensed against periodontal disease; however, several efforts are underway to develop a vaccine [20]. Herein, reverse vaccinology was integrated with subtractive proteomics to prioritize potential vaccine candidates in the proteome of oral pathogens, especially *P. gingivalis* followed by epitopes mapping using immunoinformatic techniques. Further, biophysics approaches including molecular modelling and molecular dynamics simulation approaches were employed to probe designed vaccine ensemble interactions with the innate immune receptors and understand vaccine-immune receptor dynamics in solution. We hypothesized that the vaccine will be helpful for experimentalists in vaccine development against oral pathogens. The prime significance of the study is to provide an excellent platform for vaccinologists to make use of the in silico-based vaccine in experimental in vivo and in vitro studies to disclose the real immune protection efficacy of the vaccine. This will shorten the vaccine development period and will save on the associated cost of vaccine development. From the user perspective, it will lower the burden of antibiotic resistance and improve human health in general.

## 2. Research Methodology

**Figure 1:**
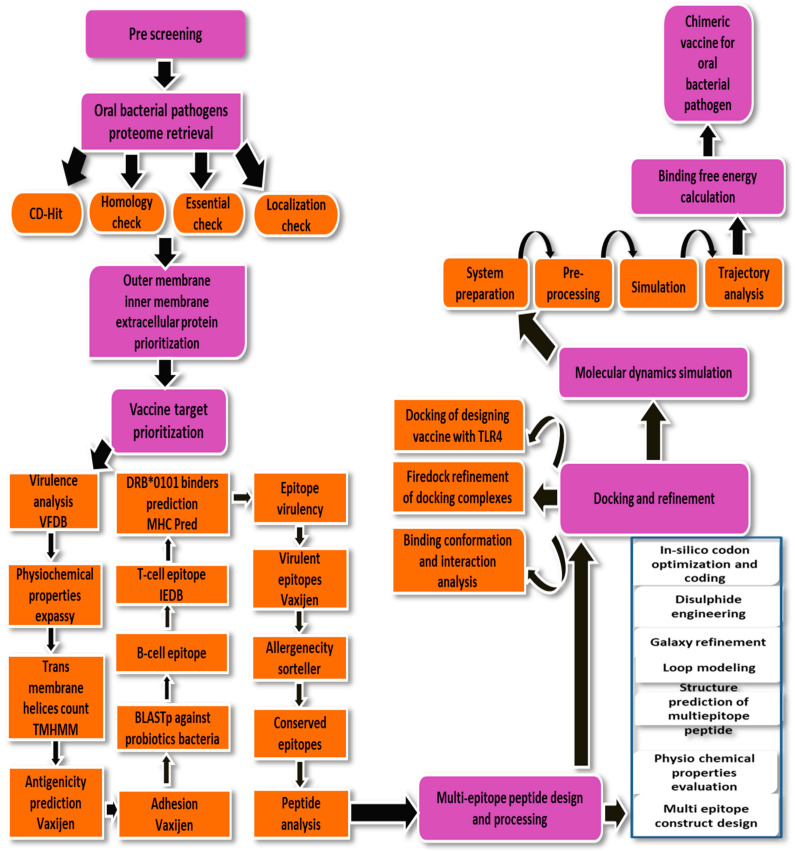
Schematic diagram of the methodology used to design a multi-epitopes vaccine construct against *P. gingivalis*.

For designing a multi-epitopes vaccine, the following methodology flow was used as given in Figure 1.

### 2.1. Subtractive Proteome and Reverse Vaccinology Phase

The data of pathogen proteomes were extracted from the genome database of NCBI [21]. At the time of the research, we retrieved three complete sequenced genomes of *P. gingivalis*. Potential vaccine candidates were identified using filters, and methods discussed in [22,23,24,25,26].

### 2.2. Pre-Screening Phase

BLASTp was considered for predicting host non-similar proteins as well as pathogen-specific proteins [27]. Essential proteins of the pathogens were identified using the database of essential genes (DEG) [28].

The screening of vaccine proteins was performed as follows; (i) conservation in the sequenced strains of the pathogen [29] (ii) not present in the human host [30], (iii) critical for the growth of bacteria [31] (iv) exposed to the host environment [25] (v) non-redundant and part of the core genome [24]. For the identification of conserved proteins, a bacterial pan-genome analysis tool was employed [32]. BLASTp was performed using different parameters, i.e., the identity of the sequence was required to be greater than 30%, an E-value smaller than 1.0 E−5, and bit score greater than 100 [33]. Using BLASTp, we checked the similarity of these sequences with normal flora, i.e., *Lactobacillus rhamnosus*, *Lactobacillus casei*, *Lactobacillus jhonsoni* and *Bacteroides* (oral normal flora) and found no similarity. Essential proteins of the pathogens were identified using the DEG database [28] and the essential proteins were those who fulfill the criteria of parameters discussed above [34].

### 2.3. Cluster Database at High Identity with Tolerance (CD-HIT) Analysis

Redundant proteins are not part of the core genome because they are not appraised as a good vaccine target [35] while, on the other hand, non-redundant proteins are known as good vaccine candidates [36]. Redundant proteins from the proteomes were discarded using CD-Hit using 50% of threshold sequence identity [37].

### 2.4. Sub-Cellular Localization Phase

Then, we analyzed the essential proteome in the subcellular localization [38] by using PSORTb 3.0 [39]. PSORTb is an online web resource commonly used to predict the subcellular localization of proteins. The proteins that are localized on the outer membrane, extracellular and periplasmic regions are regarded as good vaccine targets as they come into direct contact with the host cells and contain multiple antigenic determinants [40].

### 2.5. Vaccine Candidate’s Prioritization Phase

In this step first, surface proteins involved in pathogen disease development and progression were identified [41]. To select such proteins, BLASTp was performed against the virulent factor database (VFDB) (http://www.mgc.ac.cn/VFs/, accessed on 15 September 2022). The different parameters used in the check involved a sequence similarity check (>30%) and bit score >100 [42].

### 2.6. Physiochemical Properties Analysis

By using the online tool of ProtParam [43], physiochemical properties such as the instability index, molecular weight, theoretical PI, number of amino acids, grand average of hydropathy, and aliphatic index of selected virulent proteins were determined [43]. The proteins with a predicted value of greater than 40 were deemed unstable and discarded [40]. Similarly, proteins were considered as good vaccine targets if they had a molecular weight of smaller than 110 kDa [22].

### 2.7. Analysis of Transmembrane Helices

Selected proteins were further analyzed for transmembrane helices and only those proteins with values of 0 or 1 were selected [22,24]. Proteins with a low number of transmembrane helices are easy to purify during experimental investigation [44]. An analysis of transmembrane helices was performed using online tools named HTMMTOP [45] and TMHMM 2.0 [46].

### 2.8. Antigenicity, Allergenicity, and Adhesion Probability Prediction

Using Vaxijen [47], the antigenicity of proteins was checked [48]. Only those proteins with an antigenicity value of higher than 0.4 were selected. The allergenicity of proteins was detected using Allertop 2.0 [49]. To obtain a good vaccine candidate, adhesion was checked via Vaxign 2.0 [50].

### 2.9. Prediction of Immune Cell Epitopes

The immune epitope database (IEDB) server was used to predict B-cell epitopes and T-cell epitopes [51]. Through a server named Bepipred linear epitope 2.0 [52], B-Cell epitopes were predicted. Only the epitopes, passing the cut off score of 0.5 were selected. The T-cell epitopes were in turn predicted from the B-cell epitope using the IEDB MHC- I and MHC-II servers [22,53]. In both MHC-I and MHC-II epitopes prediction, a reference set of alleles available in the IEDB database was used. The common MHC epitopes with a low percentile score were opted for further processing.

### 2.10. MHCPred Analysis

To perform an MHCPred analysis, DRB*0101 was chosen as the receptor allele due to the highly prevalent nature of the allele in human populations [23]. In this analysis, we selected only those B-cell-derived T-cell epitopes with IC50 values smaller than 100 nm for DRB*0101 [54]. Once the final set of epitopes was finalized, the epitopes were BLASTp against *T. denticola* (tax id: 158), *A. actinomycetemcomitans* (tax id: 714), *T. forsythia* (tax id: 28112), *C. rectus* (tax id: 203) and *F nucleatum* (tax id: 851) for conservation among oral bacterial species.

### 2.11. Multi-Epitopes Vaccine Design

Peptide vaccines are weakly immunogenic which can be overcome by designing a multi-epitopes vaccine [55,56]. By using linkers such as GPGPG, antigenic epitopes can be linked with each other and to the beta-subunit of cholera toxin to form a multi-epitope vaccine construct [57]. The linker (EAAAK) was used to link the cholera beta-subunit to the N-terminus. The 3D Pro tool of the SCRATCH protein server was used to predict the designed vaccine structure [58]. Vaccine-structure modeling was performed ab initio as no appropriate template was available.

### 2.12. Loop Modeling and Vaccine Refinement

Loop modeling was performed using a Galaxy loop server [59]. Refinement was performed using the Galaxy refine server [60] which lowers the global binding energy and lowers the error in the 3D structure.

### 2.13. Disulfide Engineering and Codon Optimization

For achieving stability, disulfide bonds were introduced to the vaccine by Design 2.0 [2,61]. To ensure the maximum expression of the vaccine in *Escherichia coli*, in silico codon optimization was performed using the Java Codon Adaptation tool (JCat) [62]. A vaccine candidate with a good GC value and codon adaptation index (CAI) value can be considered to possess a good expression level in *E. coli*.

### 2.14. Docking and Refinement

In this step, designed chimeric vaccine docking was performed with immune receptors [63,64]. The vaccine was blindly docked with TLR-4 (PDB: 4G8A), MHC-I (PDB ID: 1L1Y), and MHC-II (1KG0) receptors [65] using an online server of PATCHDOCK [66]. The selection of TLR-4 was made as it plays a key role in host defense against Gram-negative bacteria. It activates the signaling pathway of NF-κB and inflammatory cytokine production that leads to the activation of innate immunity, which in turn results in adaptive immune responses against *P. gingivalis* [67,68]. The server provided 20 docking solutions and each docking solution was assigned a global binding energy. Subsequently, the complexes were refined with FireDock [69] to re-score the docked solutions after extensive refinement of the complexes. The best complex with a low global binding energy in each case was further selected for intermolecular interactions and binding conformation using UCSF Chimera.13.1 [70].

### 2.15. Molecular Dynamics Simulation

The dynamic behavior of vaccine-immune receptors can be investigated through in silico methods such as a molecular dynamics simulation. Based on the global energy value, complexes were selected for the molecular dynamic simulation phase. The analysis was performed using AMBER20 simulation software on a time scale of 200 ns. This process consists of three steps, i.e., preparation of the system, pre-processing, and production [71] and was carried out using the AMBER SANDER module [72]. The complexes were solvated into a TIP3P solvation box of 12 Angstrom padding distance [73]. The complexes were first heated to 300 K, followed by equilibration for 1 ns. This was followed by a production run of 250 ns and each trajectory file was saved at a time interval of 10 ns. The SHAKE algorithm [74] was used to constrain hydrogen bonds while Langevin dynamics was used for temperature control. The CCPTRAJ module [75] was applied for trajectories analysis while XMGRACE [76] was considered for graphs plotting.

### 2.16. MM-GBSA Binding Free Energies

Binding free energies of the vaccine-immune receptor complex were calculated using MMPBSA.py of AMBER20 [77]. About 100 frames were evaluated for free energies. The analysis estimated the difference between the binding free energies of complexes in un-solvated and solvated phases [78].

### 2.17. Immune Simulation

The designed vaccine was further characterized for an immune-response profile using the C-ImmSim server (http://150.146.2.1/C-IMMSIM/index.php, accessed on 28 September 2021), an agent based server for in silico immune system simulation in response to the vaccine antigen [79]. The server used a position-specific scoring matrix and machine learning approaches to study immune interactions. The server stimulates three compartments such as bone marrow, the thymus and lymph nodes. During the analysis, simulation parameters were treated as default. Three injections were delivered at an interval of four weeks. The time steps used were 1, 84 and 168 [80].

## 3. Results

### 3.1. Genomes Retrieval of P. gingivalis

For the development of a multi-epitope-based vaccine, we required completely sequenced genome sequences. Three completely sequenced genomes of *P. gingivalis* were retrieved from the NCBI genome database (https://www.ncbi.nlm.nih.gov/genome/714, accessed on 25 October 2021). On average, the size of pathogen strains varied from 2.34 Mb to 2.35 Mb with average GC contents of 48.5%. The net number of genes in each strain was about 1542. Table 1 explains the strain type, the genome size and percent of GC content.

### 3.2. Bacterial Pan-Genome Analysis

**Figure 2:**
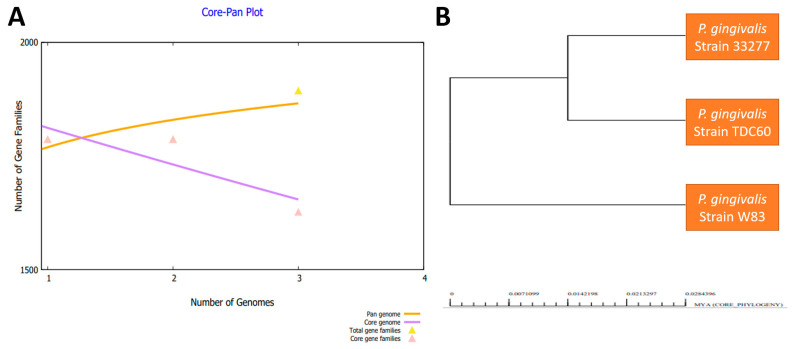
(**A**) Pan-core plot of *P. gingivalis* genomes. (**B**) Pan-phylogeny tree of *P. gingivalis* genomes.

In the next step, we performed a bacterial pan-genome analysis to obtain the core genome for downward steps. By conducting a pan-genome analysis, core genome and accessory genomes were generated [81]. The core genes are common among species genomes and are used to better understand genome evolution, gene orthology, genome complexity and the mining of pathogenic and therapeutic sequences. Pan-genome encompasses all strain genomic sequences while the core genome set is the set of sequences common in all strains. The accessory genomes represent a set of sequences present in one or more strains but not in all strains. These accessory genes are also called accessory proteomes or dispensable proteomes. Unique genes are present in only one strain and are strain-specific, also called singleton. The core genome contains those proteins which are conserved across the strains. In Figure 2A, the genome size of the strains is presented, while in Figure 2B a pan-phylogeny tree of *P. gingivalis* is provided. The number of core proteins was 4908, which contained both redundant and non-redundant sequences.

### 3.3. CD-HIT Analysis

**Figure 3:**
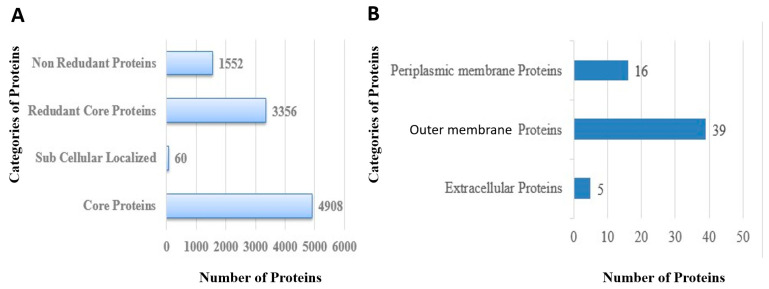
(**A**) Number of total sub cellular localized, core, redundant and non-redundant proteins, (**B**) Number of extracellular, periplasmic and outer membrane proteins.

In total, 1552 non-redundant proteins and 3356 redundant proteins were identified in the core genome of the pathogen as shown in Figure 3A. The redundant proteins were removed as they were duplicated sequences and thus not considered as good vaccine candidates. The non-redundant proteins were processed further [82].

### 3.4. Proteins Subcellular Localization

The proteins present on the surface and in the periplasmic, extracellular and outer membranes can be easily recognized by the host immune system. In total, sixty proteins were found to be present at the pathogen surface among which sixteen were periplasmic proteins, thirty nine were outer membrane proteins and five were extracellular proteins, as shown in Figure 3B.

### 3.5. VFDB Analysis

In total, six virulent proteins were identified as per the criteria defined in the Methodology Section 2. In these results, we found two selected outer membranes, three periplasmic and one extracellular membrane, as shown in Table 2. Virulent proteins can act as attractive vaccine targets as they can stimulate immune pathways resulting in the improved production of safe immune responses. As only patches of such proteins are used in vaccine design, it is safe to use them in vaccine formulations without producing any detrimental effects on human cells.

### 3.6. Transmembrane Helices and Physiochemical Analysis

In the transmembrane helices analysis, only proteins that harbored 0 or 1 transmembrane helices were selected. In this analysis, two proteins were removed from a total of six proteins. Proteins with a smaller number of transmembrane helices are easy to experimentally analyze due to their easy cloning and expression analysis. Through physiochemical analysis, one protein was discarded due to having a greater molecular weight than the threshold value. So, three target proteins of vaccines were shortlisted. In Table 3, results of the physicochemical properties analysis of all six proteins are shown.

### 3.7. Similarity with Human Genome and Prediction of Antigenicity and Allergenicity

After the physiochemical analysis of proteins, the shortlisted proteins were subjected to an homologous check. The proteins used in the designing of vaccines must not be homologous to the host as homologous proteins can cause autoimmune diseases. Only those proteins that are not homologous to the human genome were forwarded. Similarly, those proteins that were antigenic and not allergic were selected. The host non-similar, antigenic and non-allergic proteins are shown in Table 3.

### 3.8. Homology Check of Normal Flora

The selected three proteins shown in Table 4 were also found to show no homology to normal flora of human. The strains of bacteria used were *L. casei*, *L. johnsonii*, *L. rhamnosus* and *Bacteroides* (oral normal flora). This analysis helped in selecting those proteins which avoid the accidental inhibition of host normal flora.

### 3.9. B-Cell Epitopes Prediction

After passing through all essential filters required for a good vaccine candidate, the selected three proteins were subjected to the epitopes prediction phase. After which we predicted the B-Cell epitope and T-Cell epitope using the IEDB server [83]. First, B-cell epitopes were predicted. A total of nine B-cell epitopes were predicted for lytic transglycosylase domain-containing proteins, five for superoxide dismutase protein, and six for FKBP-type peptidyl-propyl cis-trans isomerase as tabulated in Table S1.

### 3.10. MHC-I and MHC-II Epitopes Prediction

The T-cell-epitope prediction phase involves MHC-I and then MHC-II binding, as described in Table S2. The MHC-I and MHC-II alleles used are tabulated in Table S3.

### 3.11. Epitope Prioritization Phase

Different filters such as MHCPred, water-solubility, toxicity, allergenicity and antigenicity were applied to the selected epitopes to prioritize those which can be used in a multi-epitopes vaccine design.

### 3.12. MHCPred, Allergenicity, Antigenicity, Solubility and Toxicity Analysis

**Figure 4:**
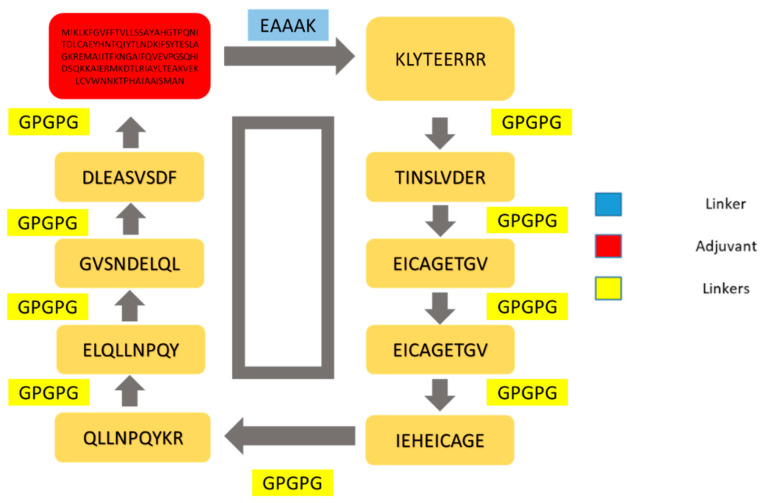
Schematic diagram of the vaccine construct. The yellow color shows the linker (GPGPG) which is used to link selected epitopes. The red color represents the adjuvant (cholera toxin B-Subunit) while the blue color represents EAAAK (linker).

By utilizing MHCPred, the binding affinity of epitopes for DRB*0101 was evaluated. Only epitopes with IC_50_ values < 100 nm were selected because DRB*0101 is the most common allele present in 95% of the population [84]. The epitopes with an IC_50_ value of smaller than 100 nm are shown in Table 5; only antigenic and non-allergic epitopes were selected to stimulate strong and safe immune responses. The antigenic and non-allergic epitopes are tabulated in Table 5. The solubility of epitopes was checked using the Protein-Sol web server [85]; the server can easily predict the solubility of a vaccine molecule and only soluble epitopes were selected [86]. Toxin-Pred was employed for the selection of non-toxic epitopes. In Table 5, epitopes that are antigenic, non-allergic, and non-toxic and have good water solubility are listed. These selected epitopes were then forwarded to make a multi-epitopes vaccine. The nine shortlisted epitopes are also schematically presented in Figure 4. Similarly, the screened epitopes were conserved in *T. denticola*, *A. actinomycetemcomitans*, *T. forsythia*, *C. rectus* and *F. nucleatum* and thus the epitopes can be used in broad-spectrum vaccine design.

### 3.13. Multi-Epitopes Vaccine Designing

**Figure 5:**
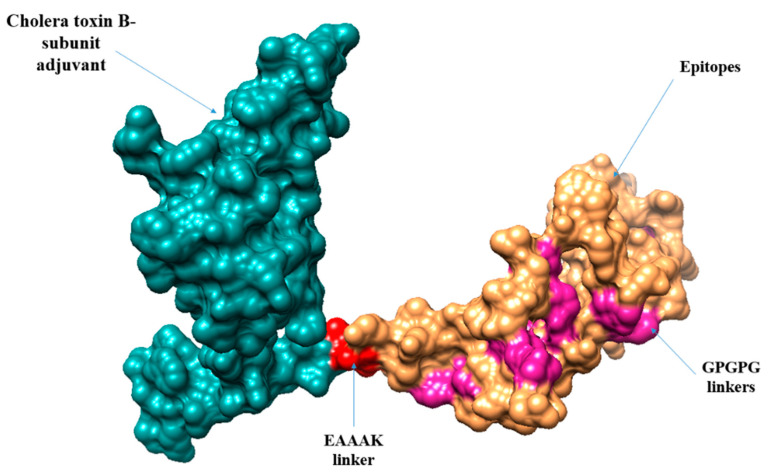
The 3D structure of vaccine construct representing GPGPG linkers, cholera toxin B subunit, EAAAK linker, and vaccine epitopes.

A multi-epitopes was designed to overcome the weak immunogenicity of epitopes [87]. The epitopes were joined with linkers to allow efficient separation of the epitopes. Additionally, an adjuvant molecule was added to the multi-epitopes peptide to further enhance the antigenic and immunogenic potential of the vaccine. The adjuvant used was cholera toxin B-subunit, which is a potent stimulator of interferons and cellular immunity. The schematic representation of the multi-epitopes vaccine construct is shown in Figure 5.

### 3.14. Vaccine Structure Prediction, Loops Modeling and Refinement

The three-dimensional structure of the vaccine construct was modeled to further understand vaccine binding with different immune receptors and the exposed nature of the vaccine epitopes. Ab initio structure modeling was performed as no appropriate template was available at the time of vaccine-structure modeling. The designed vaccine 3D structure is given in Figure 5.

To avoid structure instability, the following loop-comprising residues were modeled into secondary structure elements to obtain the most refined structure: Met1-Val8, Lys55-Pro74, Ala101-Asn111, Glu158, Gly165, Glu177, Pro184, Pro196, Leu202, Gln216, Pro220, Gln235-Pro240, Cys30-Thr36, Val149-Gly157, Val166-Pro170, Gly185, Ile190, Asn203-Lys207, Gln221-Gly228, Ser246-Phe250, Glu50-Gly54, Gly171-Gly176, Cys191-Gly195, Arg208-Leu215, Val229-Leu234.

### 3.15. Disulfide Engineering and Codon Optimization

**Figure 6:**
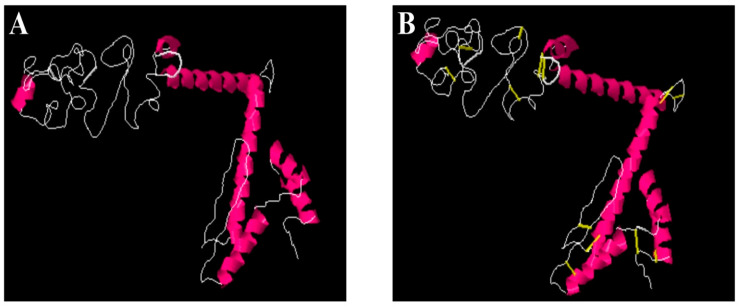
(**A**) Wild and (**B**) mutated structure of vaccine construct. The introduction of disulfide bonds is shown by yellow bands in a mutated structure.

The vaccine was further subjected to disulfide engineering to strengthen the intermolecular bonding of the vaccine and enhance vaccine structure stability. This further ensured that weaker segments of the vaccine were resistant to cellular degradation and conferred conformation stability to the vaccine [88]. During the analysis, only residue pairs with a higher energy value (>0 kcal/mol) were mutated to cysteine. The amino acid residues replaced by cysteine are tabulated in Table 6, while the cysteine bonds are shown by yellow sticks in Figure 6.

The vaccine sequence was reverse translated into a DNA sequence to perform codon optimization according to the *E. coli* expression system. The GC value of the vaccine was 57.08% and the CAI value was 0.92. Both values are indicators of a highly expressed sequence.

### 3.16. Analysis of Molecular Docking

Robust interactions of a vaccine with receptors are necessary for generating good immune responses. To analyze the interaction of the host receptors and vaccine construct, we conducted blind molecular docking. The top 20 docked solutions of vaccine with MHC-I, MHC-II, and TLR-4 were picked as shown in Tables S4–S6.

### 3.17. Docked Complexes Refinement

The docked complexes were further refined to remove false positive results and select the complex with the lowest binding energy. The term lowest binding energy complex implies the best binding of the vaccine with immune receptors. In case of MHC-I, solution number 5 was selected as it had the lowest global energy of −13.83 KJ·m^−1^. In MHC-II, solution number 2 with a global binding energy value of −11.10 KJ·m^−1^ was selected. In case of TLR-4, solution number 9 was selected as it had the lowest global energy of −13.10 KJ·m^−1^. The FireDock rescored docked solutions are given in Table 7, Table 8 and Table 9.

### 3.18. Docked Conformation of Vaccine with Immune Receptors

**Figure 7:**
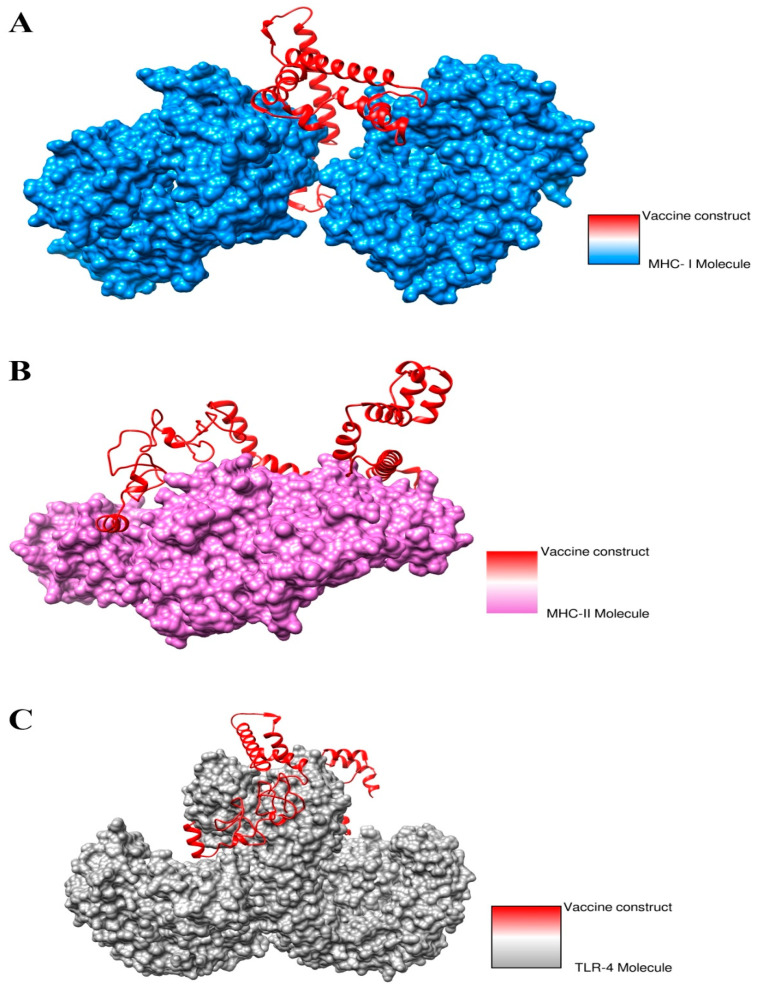
(**A**) Docked conformation of the vaccine with MHC-I molecule (**B**) Vaccine with MHC-II molecule (**C**) Vaccine with and to TLR-4 molecule.

The best docked complex for each receptor was visualized to investigate the docked conformation of the vaccine with immune receptors such as MHC-I, MHC-II and TLR-4 as shown in Figure 7. The vaccine was observed to perform deep binding with the receptors and the epitopes were exposed to the host immune system cells for recognition and processing. This further implies that the vaccine epitopes can stimulate proper immune responses, leading to the generation of humoral and cellular immunity.

### 3.19. Interactions of Vaccine to Immune Receptors

Understanding the interactions type and number of interactions between the vaccine and receptors is important as they are key in determining the strength of vaccine–receptors interactions. Different types of interactions were observed, especially hydrophilic, hydrophobic, salt bridges and di-sulfide bonds, between vaccines and receptors. All these interactions were found to play a key role in stability of the docked conformation of the vaccine with the immune receptors. These interactions require a number of residues of the receptors to engage the vaccine molecules. These residues are shown in Table 10.

### 3.20. Molecular Dynamics Simulation

**Figure 8:**
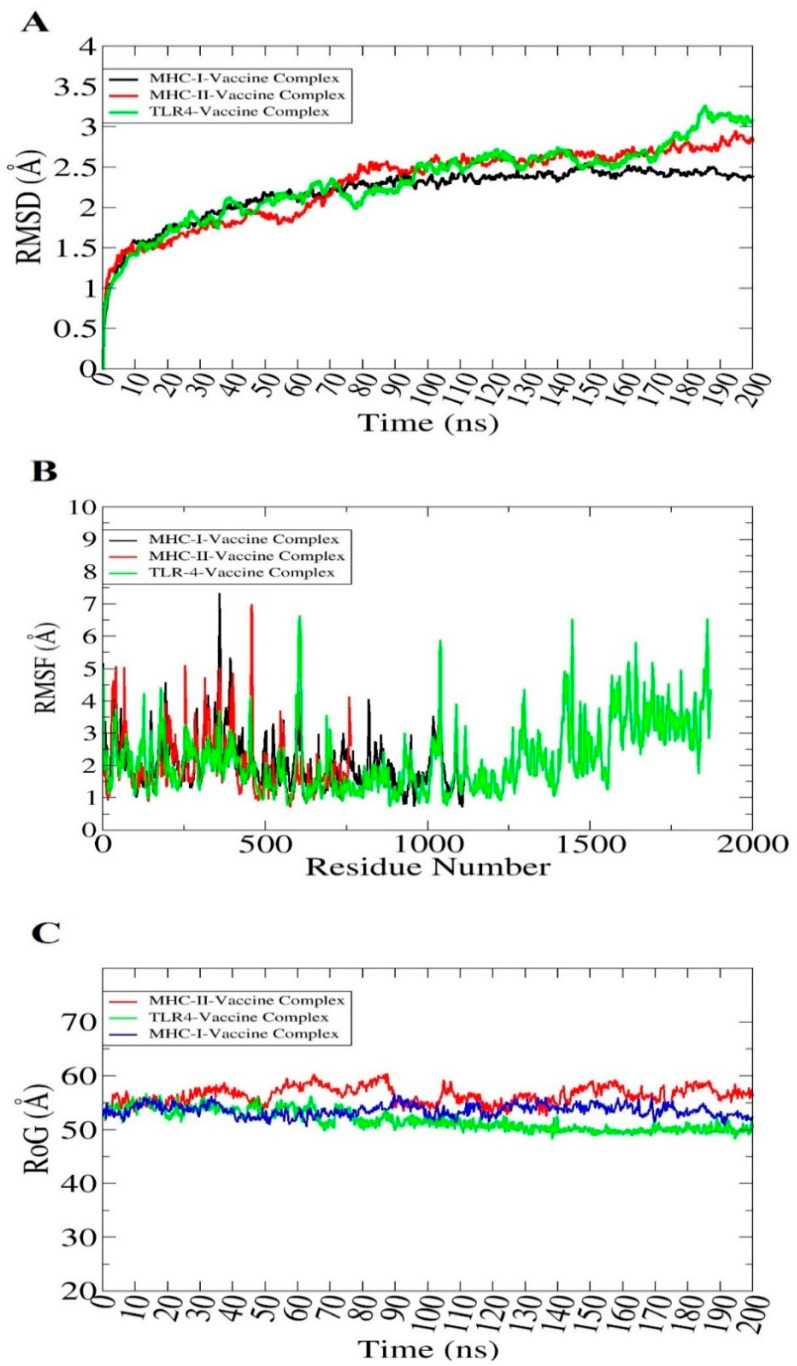
Simulation trajectories analysis. RMSD (**A**), RMSF (**B**) and RoG (**C**). The figures were generated using XMGRACE software.

The dynamic behavior of selected docked complexes was checked through an all-atom molecular dynamics simulation. The simulation trajectories were investigated through the radius of gyration (RoG), root mean square deviation (RMSD) and root means square fluctuation (RMSF) based on the carbon alpha atoms. This analysis was vital to understand the dynamic binding stability of the vaccine with respect to receptors and determine whether the epitopes were exposed to the host immune cells. The plot of RMSD remained stable from the start and no major changes were observed in the structures. Few minor structure deviations were noted that were due to the many loops present in the systems. The RMSD plot varied between 2.5–3 Å throughout the length of simulation time as shown in Figure 8A. Further, RMSF was determined, which depicted that major receptors’ residues remained stable with few high flexibilities due to the loops in the presence of the vaccine molecule. The majority of the residues present in the system were smaller than 3 Å which shows that they have better stability (Figure 8B). To further validate these findings, RoG was investigated for the systems as presented in Figure 8C. The systems were observed to have a good compact nature and the secondary structures were found to have good tight conformation. These results are in agreement with the RMSD and RMSF results and overall indicate good system stability.

### 3.21. Calculation of Binding Free Energies

Binding free energies of the docked complexes were calculated using MM-GBSA and MM-PBSA approaches. Both these approaches are well known and considered modest approaches due to high speed and good accuracy. These calculations were used to validate the binding stability of the docked complexes. The total binding free energy of the vaccine-TLR-4 complex was −135.73 kcal/mol, for the vaccine-MHC-I complex it was −101.32 kcal/mol and for the vaccine-MHC-II vaccine complex it was −76.17 kcal/mol as shown in Table 11. Electrostatic and van der Waals energies contributed positively to complex formation.

### 3.22. Immune Stimulations

**Figure 9:**
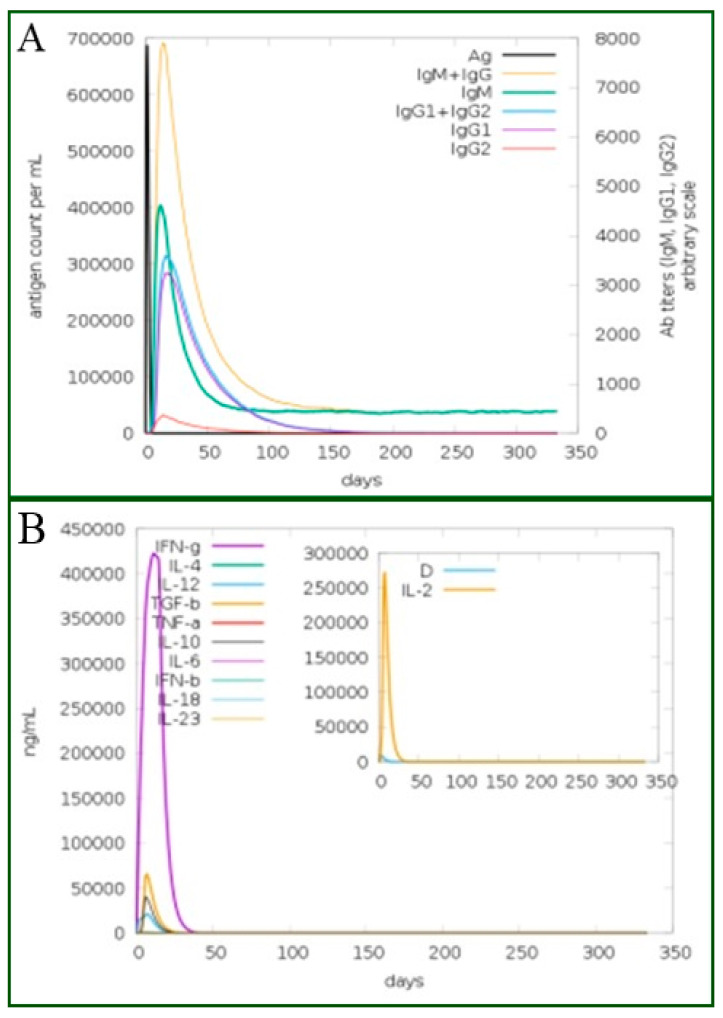
(**A**) The various types immunoglobulins, and the immunocomplexes. (**B**) The concentration of cytokines and interleukins in response to the vaccine. The inner plot box shows danger signal together with leukocyte growth factor IL.

The vaccine antigen was exposed to the host immune system for 350 days. It was revealed that an increased IgM and IgG antibodies level were observed against the antigen. The secondary response, in turn, increased the tertiary responses and resulted in the formation of B-cells, IgG1, IgG2, IgG1+IgG2, IgM and IgM+IgG as shown in Figure 9A. Similarly, in Figure 9B, the production of interferon-gamma is shown to be greater than 250,000 counts per ml. The different B-cell and T-cell responses are shown in Figures S1 and S2, respectively. The humoral and cellular immune responses were documented to play a significant role in clearing *P. gingivalis* and related oral bacterial pathogens. Innate immune cells such as dendritic cells and adaptive immunity lymphocytes, and monocytes/macrophages localized in periodontium recognize and respond to *P. gingivalis* through pattern recognition receptors (PRRs), followed by the release of inflammatory cytokines and reactive oxygen species [18]. As *P. gingivalis* is an oral pathogen, secretory IgA antibody plays a role as the first line of defense by blocking pathogen adherence to host mucosal surfaces [89].

## 4. Discussion

*P. gingivalis* is a Gram-negative anaerobic bacterium that is responsible for periodontitis which results in teeth loss. More than 500 bacterial species inhabit the human oral cavity and most of them are non-pathogenic. However, some, such as *P. gingivalis*, form biofilm that contributes to chronic periodontitis [16]. Recently, the bacterium has been reported to have an association with the development of Alzheimer’s [90]. The bacterium is resistant to multiple antibiotics such as clindamycin, metronidazole, and amoxicillin and therefore warrants the search for novel antibiotics and vaccines to manage the said bacterial pathogenicity [91].

Vaccines have excellent potential of preventing infections and proved so by saving millions of lives from many pandemics in the past. Successful examples of vaccines that saved humanity from pandemics include the Spanish flu vaccine and smallpox vaccine. The has been a significant effect of vaccine development in combatting many diseases. Traditional vaccinology, though still in use and has been successful for many decades, suffers from several limitations that shift the focus towards genome-based vaccines. The use of bioinformatics in recent times has considerably broadened the scope of vaccinology, particularly for those pathogens which are unable to culture in lab conditions and those which undergo continuous genetic changes in surface antigens. Reverse vaccinology, which is the reverse of traditional vaccinology, has now attracted more attention due to its key role in the recent development of the meningococci vaccine [92,93]. Reverse vaccinology is genome-based vaccinology and has contributed remarkably to designing of multi-epitopes vaccines [94,95,96].

Several attempts have been made to develop a vaccine against *P. gingivalis* and other oral bacterial pathogens so far. The heat shock proteins of this bacteria have been investigated for vaccine development and it has been concluded that the *P. gingivalis* HSP60 protein has the potential to reduce periodontitis in mice models [97]. In another study, PG32 and PG33 proteins were found to show immune protective efficacy and clear the pathogen [98]. Despite these efforts, none of the study findings are convincing and no appropriate vaccine candidate is under development. Considering this, herein, we performed an in-depth computational vaccine design study to thoroughly screen the core genome of *P. gingivalis* and identify proteins that are capable of stimulating host immune responses.

In this investigation, three potential vaccine targets, namely lytic transglycosylase domain-containing protein, superoxide dismutase enzyme, and FKBP-type peptidyl-propyl cis-trans isomerase enzyme were identified and fulfilled all the required potential vaccine candidate properties. The targets are part of the pathogen core genome thus ensuring the development of a broad-spectrum vaccine. Further, it was ensured that these proteins were present on the pathogen surface. Such proteins are easily accessible to the host immune system for interactions. These proteins also harbor strong antigenic determinants capable of stimulating the immune system. The selected proteins are also non-homologous to human proteomes and thereby good candidates for avoiding autoimmune responses. Moreover, the proteins are antigenic and able to bind products of acquired immunity and activate immune signaling pathways. Immunoinformatics further affirms that these proteins harbor antigenic epitopes that are non-toxic, antigenic, non-allergic, and have strong binding affinity for the DRB*0101 allele. This allele is present in the majority of the human population and the interaction of epitopes with this allele leads to accurate and robust immune responses. The predicted epitopes were further utilized in multi-epitopes vaccine design to remove the limitations of a single peptide vaccine. The designed vaccine showed stable binding conformation with different immune receptors such as MHC-I, MHC-II, and TLR-4. As the findings of the intermolecular interaction analysis revealed, multiple hydrophilic and hydrophobic interactions were formed between the vaccine and receptors, thereby leading to stable complex formation. Towards the end, the vaccine candidate was evaluated for its potential to stimulate host immune system cells. High primary, secondary, and tertiary immune responses were noticed. Similarly, a high concentration of interleukins and interferons was observed.

Computer-aided vaccine design based on genomic data is gaining rapid recognition for vaccine development. It is not only time and money saving but could deliver data in a short time for specific experimentations. All these findings suggest that the designed vaccine is a good candidate for in vivo and in vitro testing.

## 5. Conclusions and Limitations

A multi-epitopes vaccine against an oral bacterial pathogen, *P. gingivalis* and other oral bacterial pathogens, was proposed in this research using a variety of computer-aided vaccine design techniques, including reverse vaccinology, subtractive proteomics, immune-informatics, and several biophysical analyses. The vaccine epitopes were predicted using the following three potential vaccine targets: lytic transglycosylase domain-containing protein, superoxide dismutase enzyme, and FKBP-type peptidyl-propyl cis-trans isomerase enzyme. The mentioned targets were prioritized based on several vaccine candidacy parameters including but not limited to the presence of protein in the core proteome of the pathogen, being present on the cell surface, non-homologous to the host, the presence of probiotic bacteria, and being feasible for experimental analysis. Similarly, the epitopes used in the vaccine were non-toxic, antigenic, non-allergic, and had high binding potential for B-cell alleles, and T-cell alleles. The designed vaccine construct showed excellent binding with the different immune receptors and remained stable for a simulated period of time. Host immune-system simulation in response to the vaccine unveiled the production of strong primary, secondary and tertiary immune responses. All these findings determined the vaccine as a good candidate to be evaluated for its immune protection ability in in vivo models. A vaccine against *P. gingivalis* could be developed faster with our findings and data from the study might speed up the process of vaccine discovery against this pathogen. Even though our selection criteria were quite tight throughout the study, there are still some shortcomings that must be addressed in future investigations. Firstly, the ordering of epitopes in the vaccine for optimal activity was not tested. Secondly, the MHC epitopes-prediction algorithm’s accuracy was not tested extensively.

## Figures and Tables

**Table 1:** Different statistics of *P. gingivalis* completely sequence strains.

Organism Name	Strain	Size (Mb)	GC%
*P. gingivalis*	ATCC 33277	2.35	48.4
*P. gingivalis*	TDC60	2.34	48.3
*P. gingivalis*	W83	2.34	48.3

**Table 2:** Virulent proteins identified from the set of exposed proteins.

Protein ID	Bit Score	Sequence Identity
Outer membrane		
>core/451/1/Org1_Gene734	117	35%
>core/207/1/Org1_Gene488	1303	99%
Extracellular membrane		
>core/12/1/Org1_Gene1814	208	39%
Periplasmic membrane		
>core/1384/1/Org1_Gene274	213	53%
>core/351/1/Org1_Gene1345	281	39%
>core/1029/1/Org1_Gene254	112	36%

**Table 3:** Different physicochemical analysis of virulent proteins. Transmembrane helices (T.M.H), molecular weight (M.W), theoretical (T.PI), and instability index (I.I).

Vaccine Target	T.M.H		Physiochemical Properties			Human Blast	Antigenicity	Allergenicity
Extracellular membrane	HMMTOP	TMHMM	M.W	T.PI	(I.I)			
>core/12/1/Org1_Gene1814	1	0	149.58	5.13	25.75	Non-Similar	0.42	Non-allergen
Outer membrane								
>core/207/1/Org1_Gene488	5	0	73.12	8.9	8.9	Non-similar	0.54	Non-allergen
>core/451/1/Org1_Gene734	0	0	50.89	9.72	9.72	Non-similar	0.60	
Periplasmic membrane								
>core/351/1/Org1_Gene1345	2	1	52.79	6.43	19.98	−0.092	0.59	Non-allergen
>core/1029/1/Org1_Gene254	1	0	30.28	6.65	23.64	Non-similar	0.47	Non-allergen
>core/1384/1/Org1_Gene274	0	0	21.50	5.97	19.27	Non-Similar	0.50	Non-allergen

**Table 4:** Homology check analysis of vaccine targets against selected probiotic bacterial species.

S.NO	Shortlisted Proteins	*L. casei*	*L. johnsonii*	*L. rhamnosus*	*Bacteroides*
1	>core/451/1/Org1_Gene734 (Lytic transglycosylase domain-containing protein)	No Similarity Found			
2	>core/1029/1/Org1_Gene254 (FKBP-type peptidyl-propyl cis-trans isomerase)				
3	>core/1384/1/Org1_Gene274 (Superoxide dismutase protein)				

**Table 5:** Shortlisted epitopes after MHC-Pred, antigenicity, allergenicity, solubility, and toxin-pred analysis.

Selected Epitopes	DRB*01 01 IC_50_ Score	Antigenicity	Allergenicity	Solubility	Toxin-Pred
KLYTEERRR	12.11	0.1368	Non-allergen	Good water soluble	Non-toxin
TINSLVDER	9.95	0.6485			
EICAGETGV	93.76	0.8637			
EICAGETGV	93.76	0.8637			
IEHEICAGE	52.48	0.6314			
QLLNPQYKR	10.26	0.574			
ELQLLNPQY	19.54	1.2269			
GVSNDELQL	27.35	1.3654			
DLEASVSDF	17.14	0.687			

**Table 6:** Sequence number (S.N), Chi value, Energy, and sum B-Factors of amino acid (A.A).

S. N	A. A	Sequence Number	A. A	Chi3	Energy	Sum B-Factors
6	Phe	34	His	103.11	4.15	0
57	Glu	69	Phe	111.41	7.45	0
74	Pro	77	Gln	117.49	3.97	0
104	Glu	110	Asn	94.29	2.09	0
104	Glu	113	Thr	98.57	4.81	0
104	Glu	114	Pro	89.56	4.62	0
150	Asp	154	Pro	84.4	4.89	0

**Table 7:** FireDock solutions of MHC-I-vaccine. KJ·m^−1^ is the unit of energy for each term given below.

Rank	Solution Number	Global Energy	Attractive van der Waals	Repulsive van der Waals	Atomic Contact Energy	Hydrogen Bonds Energy
1	5	−13.83	−7.21	2.90	−5.95	−0.81
2	9	−5.42	−32.42	25.47	10.50	−1.38
3	1	1.38	−0.17	0.00	0.37	0.00
4	7	15.35	−0.04	0.00	0.45	0.00
5	2	28.20	−7.73	6.54	6.57	−0.93
6	3	39.32	−11.69	46.34	4.65	−2.39
7	6	62.01	−21.28	93.15	6.42	−5.06
8	4	86.39	−25.68	125.90	7.34	−3.11
9	10	236.35	−25.31	300.30	5.74	−2.77
10	8	1229.01	−57.59	1598.19	3.99	−5.68

**Table 8:** FireDock solutions of MHC-II-vaccine. KJ·m^−1^ is the unit of energy for each term given below.

Rank	Solution Number	Global Energy	Attractive van der Waals	Repulsive van der Waals	Atomic Contact Energy	Hydrogen Bonds Energy
1	2	11.10	−1.42	0.26	−0.41	0.00
2	3	77.74	−20.90	84.35	16.05	−1.80
3	8	275.98	−34.08	352.40	8.64	−1.46
4	9	283.33	−25.69	372.45	18.43	−3.55
5	7	515.10	−9.49	643.79	3.88	−0.66
6	5	2625.03	−43.38	3325.36	13.08	−3.76
7	4	3591.01	−54.69	4575.96	3.90	−2.12
8	10	4292.57	−74.36	5482.30	5.14	−10.62
9	6	5698.37	−73.32	7235.58	12.17	−7.72
10	1	6855.32	−82.75	8713.45	8.65	−11.19

**Table 9:** FireDock solutions of TLR-4-vaccine. KJ·m^−1^ is the unit of energy for each term given below.

Rank	Solution Number	Global Energy	Attractive van der Waals	Repulsive van der Waals	Atomic Contact Energy	Hydrogen Bonds Energy
1	9	−13.10	−25.20	9.57	11.76	−3.90
2	10	5.35	−0.18	0.00	0.06	0.00
3	1	79.08	−35.14	152.47	2.38	−3.03
4	3	998.05	−36.87	1285.61	20.01	−3.89
5	5	1020.25	−34.41	1304.89	21.14	−9.75
6	2	3071.74	−70.38	3963.53	11.31	−10.56
7	7	3176.30	−74.94	4102.83	14.27	−10.73
8	8	4986.99	−83.86	6372.21	10.54	−11.68
9	6	5724.53	−49.93	7242.80	0.76	−12.72
10	4	11,424.45	−116.65	14,451.06	29.22	−14.28

**Table 10:** Interacting residues of receptors with vaccine molecule.

Vaccine Complex	Interactive Residues
MHC-I	Ala128, Ala135, Arg157, Arg181, Arg51, Asn155, Asp76, Asp129, Asp 83, Asp238, Glu128, Glu148, Glu154, Gln120, Gly79, His151, Leu23, Lys64, Lys 104, Lys144, Lys197, Phe22, Phe152, Pro50, Ser207, Ser132, Thr240, Thr240, Val9, Val152,
MHC-II	Arg44, Asn19, Asp66, Arg189, Cys174, Glu4, Glu10, Glu22, Glu187, Glu214, Gln10, Gln92, Gln174, Gly20, Leu8, Leu11, Leu45, Leu215, Lys197, Lys93, Pro183, Pro124, Pro142, Ser182, Ser126, Thr185,Thr100, Lys98, Thr21, Thr172, Tyr83,Thr181, Val99, Val91,Val86
TLR-4	Asp428, Asn464, Asn464, Asn6, Asn86, Arg606, Cys542, Leu485, Gln588,Gln588, Gln484, Gln510, Glu136, Glu509, Glu605, Glu485, Gly183, His529, His555, His557, Len462, Lys 4, Lys244, Lys560, Leu87, Phe228, Phe 463, Phe487, Phe538, Pro88, Pro489, Ser569, Tyr79, Thr548, Thr584, Val461

**Table 11:** Different binding free energies between vaccine and receptors. All values are given in kcal/mol.

Energy Parameter	TLR-4-Vaccine Complex	MHC-I-Vaccine Complex	MHC-II-Vaccine Complex
MM-GBSA			
VDWAALS	−90.14	−80.96	−70.46
EEL	−85.88	−59.00	−42.26
Delta G gas	−176.02	−139.96	−112.72
Delta G solv	40.29	38.64	36.55
Delta Total	−135.73	−101.32	−76.17
MM-PBSA			
VDWAALS	−90.14	−80.96	−70.46
EEL	−85.88	−59.00	−42.26
Delta G gas	−176.02	−139.96	−112.72
Delta G solv	43.16	39.87	42.59
Delta Total	−132.86	−100.09	−70.13

VDWAALS (van der Waals), EEL (electrostatic), Delta G gas (net gas phase energy), Delta G Solv (net solvation energy), Delta Total (net energy of the system).

## Data Availability

The data presented in this study are available within the article.

## Supplementary Materials

The following supporting information can be downloaded at: https://www.mdpi.com/article/10.3390/ijerph19148408/s1, Figure S1: Different B-cell counts in response to the antigen. Legend: Act = active, Intern = internalized the Ag, Pres II = presenting on MHC II, Dup = in the mitotic cycle, Anergic = anergic, Resting = not active; Figure S2. Different T-cell responses to the vaccine; cytotoxic killer T-cells (Tc), natural killer cells (NK), dendritic cells (DC), macrophages (Mφ), and epithelial cells; Table S1: Predicted B-cell epitopes for shortlisted vaccine targets; Table S2. Prediction of MHC-I and MHC-II epitopes from B-Cell epitopes; Table S3. MHC alleles used for epitopes prediction; Table S4. Top 20 docked solutions of vaccine for MHC-I. Table S5. Top 20 docked solutions of vaccine for MHC-II. Table S6. Top 20 docked solutions of vaccine for TLR-4.

## References

[1] Caniça M., Manageiro V., Abriouel H., Moran-Gilad J., Franz C.M. (2018). Antibiotic resistance in foodborne bacteria. Trends Food Sci. Technol..

[2] MacLean R.C., San Millan A. (2019). The evolution of antibiotic resistance. Science.

[3] Cassini A., Högberg L.D., Plachouras D., Quattrocchi A., Hoxha A., Simonsen G.S., Colomb-Cotinat M., Kretzschmar M.E., Devleesschauwer B., Cecchini M. (2019). Attributable deaths and disability-adjusted life-years caused by infections with antibiotic-resistant bacteria in the EU and the European Economic Area in 2015, a population-level modelling analysis. Lancet Infect. Dis..

[4] Bank W. (2017). Drug-Resistant Infections: A Threat to Our Economic Future.

[5] O’Neill J. (2016). Review on antimicrobial resistance: Tackling drug-resistant infections globally: Final report and recommendations. Review on Antimicrobial Resistance: Tackling Drug-Resistant Infections Globally: Final Report and Recommendations.

[6] Klemm E.J., Wong V.K., Dougan G. (2018). Emergence of dominant multidrug-resistant bacterial clades: Lessons from history and whole-genome sequencing. Proc. Natl. Acad. Sci. USA.

[7] The White House (2015). National Action Plan for Combating Antibiotic-Resistant Bacteria.

[8] National Institutes of Health (2014). NIAID’s Antibacterial Resistance Program: Current Status and Future Directions.

[9] Gagneux-Brunon A., Lucht F., Launay O., Berthelot P., Botelho-Nevers E. (2018). Vaccines for healthcare-associated infections: Present, future, and expectations. Expert Rev. Vaccines.

[10] Brooks B.D., Brooks A.E. (2014). Therapeutic strategies to combat antibiotic resistance. Adv. Drug Deliv. Rev..

[11] Ventola C.L. (2015). The antibiotic resistance crisis: Part 2, management strategies and new agents. Pharm. Ther..

[12] The White House (2014). National Strategy for Combating Antibiotic Resistant Bacteria.

[13] Reddick L.E., Alto N.M. (2014). Bacteria Fighting Back: How Pathogens Target and Subvert the Host Innate Immune System. Mol. Cell.

[14] Qamar M.T.U., Ahmad S., Fatima I., Ahmad F., Shahid F., Naz A., Abbasi S.W., Khan A., Mirza M.U., Ashfaq U.A. (2021). Designing multi-epitope vaccine against Staphylococcus aureus by employing subtractive proteomics, reverse vaccinology and immuno-informatics approaches. Comput. Biol. Med..

[15] Bloom D.E., Black S., Salisbury D., Rappuoli R. (2018). Antimicrobial resistance and the role of vaccines. Proc. Natl. Acad. Sci. USA.

[16] Manonmanipavithra R., Ari G., Rajendran S., Mahendra J. (2020). An Overview On Porphyromonas Gingivalis—An Important Periodontopathic Pathogen. Ann. Rom. Soc. Cell Biol..

[17] Gibson F.C., Genco C.A. (2001). Prevention of *Porphyromonas gingivalis*-Induced Oral Bone Loss following Immunization with Gingipain R1. Infect. Immun..

[18] Khalaf H., Palm E., Bengtsson T. (2017). Cellular Response Mechanisms in Porphyromonas gingivalis Infection.

[19] Carrouel F., Viennot S., Santamaria J., Veber P., Bourgeois D. (2016). Quantitative molecular detection of 19 major pathogens in the interdental biofilm of periodontally healthy young adults. Front. Microbiol..

[20] Vaernewyck V., Arzi B., Sanders N.N., Cox E., Devriendt B. (2021). Mucosal Vaccination Against Periodontal Disease: Current Status and Opportunities. Front. Immunol..

[21] Coordinators, NCBI Resource (2017). Database resources of the national center for biotechnology information. Nucleic Acids Res..

[22] Naz A., Awan F.M., Obaid A., Muhammad S.A., Paracha R.Z., Ahmad J., Ali A. (2015). Identification of putative vaccine candidates against Helicobacter pylori exploiting exoproteome and secretome: A reverse vaccinology based approach. Infect. Genet. Evol..

[23] Hassan A., Naz A., Obaid A., Paracha R.Z., Naz K., Awan F.M., Muhmmad S.A., Janjua H.A., Ahmad J., Ali A. (2016). Pangenome and immuno-proteomics analysis of Acinetobacter baumannii strains revealed the core peptide vaccine targets. BMC Genom..

[24] Baseer S., Ahmad S., Ranaghan K.E., Azam S.S. (2017). Towards a peptide-based vaccine against Shigella sonnei: A subtractive reverse vaccinology based approach. Biologicals.

[25] Jaiswal A.K., Tiwari S., Jamal S.B., Barh D., Azevedo V., Soares S.C. (2017). An In Silico Identification of Common Putative Vaccine Candidates against Treponema pallidum: A Reverse Vaccinology and Subtractive Genomics Based Approach. Int. J. Mol. Sci..

[26] Naz K., Naz A., Ashraf S.T., Rizwan M., Ahmad J., Baumbach J., Ali A. (2019). PanRV: Pangenome-reverse vaccinology approach for identifications of potential vaccine candidates in microbial pangenome. BMC Bioinform..

[27] Blast N. (2015). Basic Local Alignment Search Tool.

[28] Zhang R., Ou H., Zhang C. (2004). DEG: A database of essential genes. Nucleic Acids Res..

[29] Sanober G., Ahmad S., Azam S.S. (2017). Identification of plausible drug targets by investigating the druggable genome of MDR Staphylococcus epidermidis. Gene Rep..

[30] Ali A., Naz A., Soares S.C., Bakhtiar M., Tiwari S., Hassan S.S., Hanan F., Ramos R., Pereira U., Barh D. (2015). Pan-genome analysis of human gastric pathogen H. pylori: Comparative genomics and pathogenomics approaches to identify regions associated with pathogenicity and prediction of potential core therapeutic targets. BioMed Res. Int..

[31] Johri S., Solanki J., Cantu V.A., Fellows S.R., Edwards R.A., Moreno I., Vyas A., Dinsdale E.A. (2019). ‘Genome skimming’ with the MinION hand-held sequencer identifies CITES-listed shark species in India’s exports market. Sci. Rep..

[32] Chaudhari N.M., Gupta V., Dutta C. (2016). BPGA- an ultra-fast pan-genome analysis pipeline. Sci. Rep..

[33] Rizwan M., Naz A., Ahmad J., Naz K., Obaid A., Parveen T., Ahsan M., Ali A. (2017). VacSol: A high throughput in silico pipeline to predict potential therapeutic targets in prokaryotic pathogens using subtractive reverse vaccinology. BMC Bioinform..

[34] Azam S.S., Shamim A. (2014). An insight into the exploration of druggable genome of Streptococcus gordonii for the identification of novel therapeutic candidates. Genomics.

[35] Sikic K., Carugo O. (2010). Protein sequence redundancy reduction: Comparison of various method. Bioinformation.

[36] Butt A.M., Tahir S., Nasrullah I., Idrees M., Lu J., Tong Y. (2012). Mycoplasma genitalium: A comparative genomics study of metabolic pathways for the identification of drug and vaccine targets. Infect. Genet. Evol..

[37] Fu L., Niu B., Zhu Z., Wu S., Li W. (2012). CD-HIT: Accelerated for clustering the next-generation sequencing data. Bioinformatics.

[38] Ahmad S., Ranaghan K.E., Azam S.S. (2019). Combating tigecycline resistant Acinetobacter baumannii: A leap forward towards multi-epitope based vaccine discovery. Eur. J. Pharm. Sci..

[39] Yu N.Y., Wagner J.R., Laird M.R., Melli G., Rey S., Lo R., Dao P., Sahinalp S.C., Ester M., Foster L.J. (2010). PSORTb 3.0, improved protein subcellular localization prediction with refined localization subcategories and predictive capabilities for all prokaryotes. Bioinformatics.

[40] Barh D., Barve N., Gupta K., Chandra S., Jain N., Tiwari S., Leon-Sicairos N., Canizalez-Roman A., Santos A., Hassan S.S. (2013). Exoproteome and Secretome Derived Broad Spectrum Novel Drug and Vaccine Candidates in Vibrio cholerae Targeted by Piper betel Derived Compounds. PLoS ONE.

[41] Rashid M.I., Naz A., Ali A., Andleeb S. (2017). Prediction of vaccine candidates against Pseudomonas aeruginosa: An integrated genomics and proteomics approach. Genomics.

[42] Nain Z., Abdullah F., Rahman M.M., Karim M.M., Khan S.A., Bin Sayed S., Mahmud S., Rahman S.M.R., Sheam M., Haque Z. (2020). Proteome-wide screening for designing a multi-epitope vaccine against emerging pathogen *Elizabethkingia anophelis* using immunoinformatic approaches. J. Biomol. Struct. Dyn..

[43] ProtParam E (2017). ExPASy-ProtParam Tool. https://web.expasy.org/protparam/.

[44] Sajjad R., Ahmad S., Azam S.S. (2019). In silico screening of antigenic B-cell derived T-cell epitopes and designing of a multi-epitope peptide vaccine for Acinetobacter nosocomialis. J. Mol. Graph. Model..

[45] Saidijam M., Azizpour S., Patching S.G. (2017). Comprehensive analysis of the numbers, lengths and amino acid compositions of transmembrane helices in prokaryotic, eukaryotic and viral integral membrane proteins of high-resolution structure. J. Biomol. Struct. Dyn..

[46] Chen Y., Yu P., Luo J., Jiang Y. (2003). Secreted protein prediction system combining CJ-SPHMM, TMHMM, and PSORT. Mamm. Genome.

[47] Doytchinova I.A., Flower D.R. (2007). VaxiJen: A server for prediction of protective antigens, tumour antigens and subunit vaccines. BMC Bioinform..

[48] Wadood A., Jamal A., Riaz M., Khan A., Uddin R., Jelani M., Azam S.S. (2018). Subtractive genome analysis for in silico identification and characterization of novel drug targets in Streptococcus pneumonia strain JJA. Microb. Pathog..

[49] Dimitrov I., Bangov I., Flower D.R., Doytchinova I. (2014). AllerTOP v. 2—A server for in silico prediction of allergens. J. Mol. Model..

[50] He Y., Xiang Z., Mobley H.L.T. (2010). Vaxign: The First Web-Based Vaccine Design Program for Reverse Vaccinology and Applications for Vaccine Development. J. Biomed. Biotechnol..

[51] Vita R., Mahajan S., Overton J.A., Dhanda S.K., Martini S., Cantrell J.R., Wheeler D.K., Sette A., Peters B. (2018). The Immune Epitope Database (IEDB): 2018 update. Nucleic Acids Res..

[52] Jespersen M.C., Peters B., Nielsen M., Marcatili P. (2017). BepiPred-2.0, improving sequence-based B-cell epitope prediction using conformational epitopes. Nucleic Acids Res..

[53] Vashi Y., Jagrit V., Kumar S. (2020). Understanding the B and T cell epitopes of spike protein of severe acute respiratory syndrome coronavirus-2, A computational way to predict the immunogens. Infect. Genet. Evol..

[54] Ismail S., Ahmad S., Azam S.S. (2020). Vaccinomics to design a novel single chimeric subunit vaccine for broad-spectrum immunological applications targeting nosocomial Enterobacteriaceae pathogens. Eur. J. Pharm. Sci..

[55] Li W., Joshi M.D., Singhania S., Ramsey K.H., Murthy A.K. (2014). Peptide Vaccine: Progress and Challenges. Vaccines.

[56] Skwarczynski M., Toth I. (2016). Peptide-based synthetic vaccines. Chem. Sci..

[57] Nezafat N., Karimi Z., Eslami M., Mohkam M., Zandian S., Ghasemi Y. (2016). Designing an efficient multi-epitope peptide vaccine against Vibrio cholerae via combined immunoinformatics and protein interaction based approaches. Comput. Biol. Chem..

[58] Cheng J., Randall A.Z., Sweredoski M.J., Baldi P. (2005). SCRATCH: A protein structure and structural feature prediction server. Nucleic Acids Res..

[59] Giardine B., Riemer C., Hardison R.C., Burhans R., Elnitski L., Shah P., Zhang Y., Blankenberg D., Albert I., Taylor J. (2005). Galaxy: A platform for interactive large-scale genome analysis. Genome Res..

[60] Heo L., Park H., Seok C. (2013). GalaxyRefine: Protein structure refinement driven by side-chain repacking. Nucleic Acids Res..

[61] Dombkowski A.A., Sultana K.Z., Craig D.B. (2014). Protein disulfide engineering. FEBS Lett..

[62] Grote A., Hiller K., Scheer M., Münch R., Nörtemann B., Hempel D.C., Jahn D. (2005). JCat: A novel tool to adapt codon usage of a target gene to its potential expression host. Nucleic Acids Res..

[63] Morris G.M., Lim-Wilby M. (2008). Molecular docking. Molecular Modeling of Proteins.

[64] Solanki V., Tiwari M., Tiwari V. (2019). Prioritization of potential vaccine targets using comparative proteomics and designing of the chimeric multi-epitope vaccine against Pseudomonas aeruginosa. Sci. Rep..

[65] Ohto U., Yamakawa N., Akashi-Takamura S., Miyake K., Shimizu T. (2012). Structural Analyses of Human Toll-like Receptor 4 Polymorphisms D299G and T399I. J. Biol. Chem..

[66] Schneidman-Duhovny D., Inbar Y., Nussinov R., Wolfson H.J. (2005). PatchDock and SymmDock: Servers for rigid and symmetric docking. Nucleic Acids Res..

[67] Takehara M., Kobayashi K., Nagahama M. (2021). Toll-Like Receptor 4 Protects Against Clostridium perfringens Infection in Mice. Front. Cell. Infect. Microbiol..

[68] Mukherjee S., Karmakar S., Babu S.P.S. (2016). TLR2 and TLR4 mediated host immune responses in major infectious diseases: A review. Braz. J. Infect. Dis..

[69] Andrusier N., Nussinov R., Wolfson H.J. (2007). FireDock: Fast interaction refinement in molecular docking. Proteins Struct. Funct. Bioinform..

[70] Pettersen E.F., Goddard T.D., Huang C.C., Couch G.S., Greenblatt D.M., Meng E.C., Ferrin T.E. (2004). UCSF Chimera?—A visualization system for exploratory research and analysis. J. Comput. Chem..

[71] Andleeb S., Imtiaz-Ud-Din I.-U., Rauf M.K., Azam S.S., Badshah A., Sadaf H., Raheel A., Tahir M.N., Raza S. (2016). A one-pot multicomponent facile synthesis of dihydropyrimidin-2(1H)-thione derivatives using triphenylgermane as a catalyst and its binding pattern validation. RSC Adv..

[72] Case D.A., Cerutti D.S., Cheateham T.E., Darden T.A., Duke R.E., Giese T.J., Gohlke H., Goetz A.W., Greene D., Homeyer N. (2016). AMBER16 Package.

[73] Brice A.R., Dominy B.N. (2013). Examining Electrostatic Influences on Base-Flipping: A Comparison of TIP3P and GB Solvent Models. Commun. Comput. Phys..

[74] Kräutler V., Van Gunsteren W.F., Hünenberger P.H. (2001). A fast SHAKE algorithm to solve distance constraint equations for small molecules in molecular dynamics simulations. J. Comput. Chem..

[75] Roe D.R., Cheatham T.E. (2013). PTRAJ and CPPTRAJ: Software for processing and analysis of molecular dynamics trajectory data. J. Chem. Theory Comput..

[76] Turner P.J. (2005). XMGRACE, Version 5.1. 19.

[77] Miller B.R., McGee T.D., Swails J.M., Homeyer N., Gohlke H., Roitberg A.E. (2012). *MMPBSA.py*: An Efficient Program for End-State Free Energy Calculations. J. Chem. Theory Comput..

[78] Genheden S., Ryde U. (2015). The MM/PBSA and MM/GBSA methods to estimate ligand-binding affinities. Expert Opin. Drug Discov..

[79] Rapin N., Lund O., Bernaschi M., Castiglione F. (2010). Computational Immunology Meets Bioinformatics: The Use of Prediction Tools for Molecular Binding in the Simulation of the Immune System. PLoS ONE.

[80] Shey R.A., Ghogomu S.M., Esoh K.K., Nebangwa N.D., Shintouo C.M., Nongley N.F., Asa B.F., Ngale F.N., Vanhamme L., Souopgui J. (2019). In-silico design of a multi-epitope vaccine candidate against onchocerciasis and related filarial diseases. Sci. Rep..

[81] Yadav S., Kapley A. (2021). Antibiotic resistance: Global health crisis and metagenomics. Biotechnol. Rep..

[82] Uddin R., Jamil F. (2018). Prioritization of potential drug targets against P. aeruginosa by core proteomic analysis using computational subtractive genomics and Protein-Protein interaction network. Comput. Biol. Chem..

[83] Raoufi E., Hemmati M., Eftekhari S., Khaksaran K., Mahmodi Z., Farajollahi M.M., Mohsenzadegan M. (2019). Epitope Prediction by Novel Immunoinformatics Approach: A State-of-the-art Review. Int. J. Pept. Res. Ther..

[84] Albagi S., Ahmed O.H., Gumaa M.A., Abd_elrahman K.A., Abu-Haraz A.H. (2017). Immunoinformatics-Peptide Driven Vaccine and In silico Modeling for Duvenhage Rabies Virus Glycoprotein G. J. Clin. Cell. Immunol..

[85] Hebditch M., Carballo-Amador M.A., Charonis S., Curtis R., Warwicker J. (2017). Protein–Sol: A web tool for predicting protein solubility from sequence. Bioinformatics.

[86] Abraham Peele K., Srihansa T., Krupanidhi S., Ayyagari V.S., Venkateswarulu T.C. (2021). Design of multi-epitope vaccine candidate against SARS-CoV-2, A in-silico study. J. Biomol. Struct. Dyn..

[87] Javadi M., Oloomi M., Bouzari S. (2021). In Silico Design of a Poly-epitope Vaccine for Urinary Tract Infection Based on Conserved Antigens by Modern Vaccinology. Int. J. Pept. Res. Ther..

[88] Zakeri B., Fierer J.O., Celik E., Chittock E.C., Schwarz-Linek U., Moy V.T., Howarth M. (2012). Peptide tag forming a rapid covalent bond to a protein, through engineering a bacterial adhesin. Proc. Natl. Acad. Sci. USA.

[89] Kataoka K., Kawabata S., Koyanagi K., Hashimoto Y., Miyake T., Fujihashi K. (2021). Respiratory FimA-Specific Secretory IgA Antibodies Upregulated by DC-Targeting Nasal Double DNA Adjuvant Are Essential for Elimination of *Porphyromonas gingivalis*. Front. Immunol..

[90] Dominy S.S., Lynch C., Ermini F., Benedyk M., Marczyk A., Konradi A., Nguyen M., Haditsch U., Raha D., Griffin C. (2019). *Porphyromonas gingivalis* in Alzheimer’s disease brains: Evidence for disease causation and treatment with small-molecule inhibitors. Sci. Adv..

[91] Kulik E.M., Thurnheer T., Karygianni L., Walter C., Sculean A., Eick S. (2019). Antibiotic Susceptibility Patterns of *Aggregatibacter actinomycetemcomitans* and *Porphyromonas gingivalis* Strains from Different Decades. Antibiotics.

[92] Plotkin S. (2014). History of vaccination. Proc. Natl. Acad. Sci. USA.

[93] Lombard M., Pastoret P.-P., Moulin A. (2007). A brief history of vaccines and vaccination. OIE Rev. Sci. Tech..

[94] Enayatkhani M., Hasaniazad M., Faezi S., Gouklani H., Davoodian P., Ahmadi N., Einakian M.A., Karmostaji A., Ahmadi K. (2021). Reverse vaccinology approach to design a novel multi-epitope vaccine candidate against COVID-19, an *in silico* study. J. Biomol. Struct. Dyn..

[95] Tahir ul Qamar M., Shokat Z., Muneer I., Ashfaq U.A., Javed H., Anwar F., Bari A., Zahid B., Saari N. (2020). Multiepitope-Based Subunit Vaccine Design and Evaluation against Respiratory Syncytial Virus Using Reverse Vaccinology Approach. Vaccines.

[96] Bruno L., Cortese M., Rappuoli R., Merola M. (2015). Lessons from Reverse Vaccinology for viral vaccine design. Curr. Opin. Virol..

[97] Lee J.-Y., Yi N.-N., Kim U.-S., Choi J.-S., Kim S.-J., Choi J.-I. (2006). *Porphyromonas gingivalis* heat shock protein vaccine reduces the alveolar bone loss induced by multiple periodontopathogenic bacteria. J. Periodontal Res..

[98] Ross B.C., Czajkowski L., Hocking D., Margetts M., Webb E., Rothel L., Patterson M., Agius C., Camuglia S., Reynolds E. (2001). Identification of vaccine candidate antigens from a genomic analysis of *Porphyromonas gingivalis*. Vaccine.

