# Supplementary material for: Pan-Genome Analysis of Oral Bacterial Pathogens to Predict a Potential Novel Multi-Epitopes Vaccine Candidate
Source: Int J Environ Res Public Health. 2022 Jul 9;19(14):8408. doi: 10.3390/ijerph19148408 (PMC9320593; doi:10.3390/ijerph19148408)
Supplement: Supplementary file 1 [file ijerph-19-08408-s001.zip › ijerph-1686615-Supplementary.pdf]

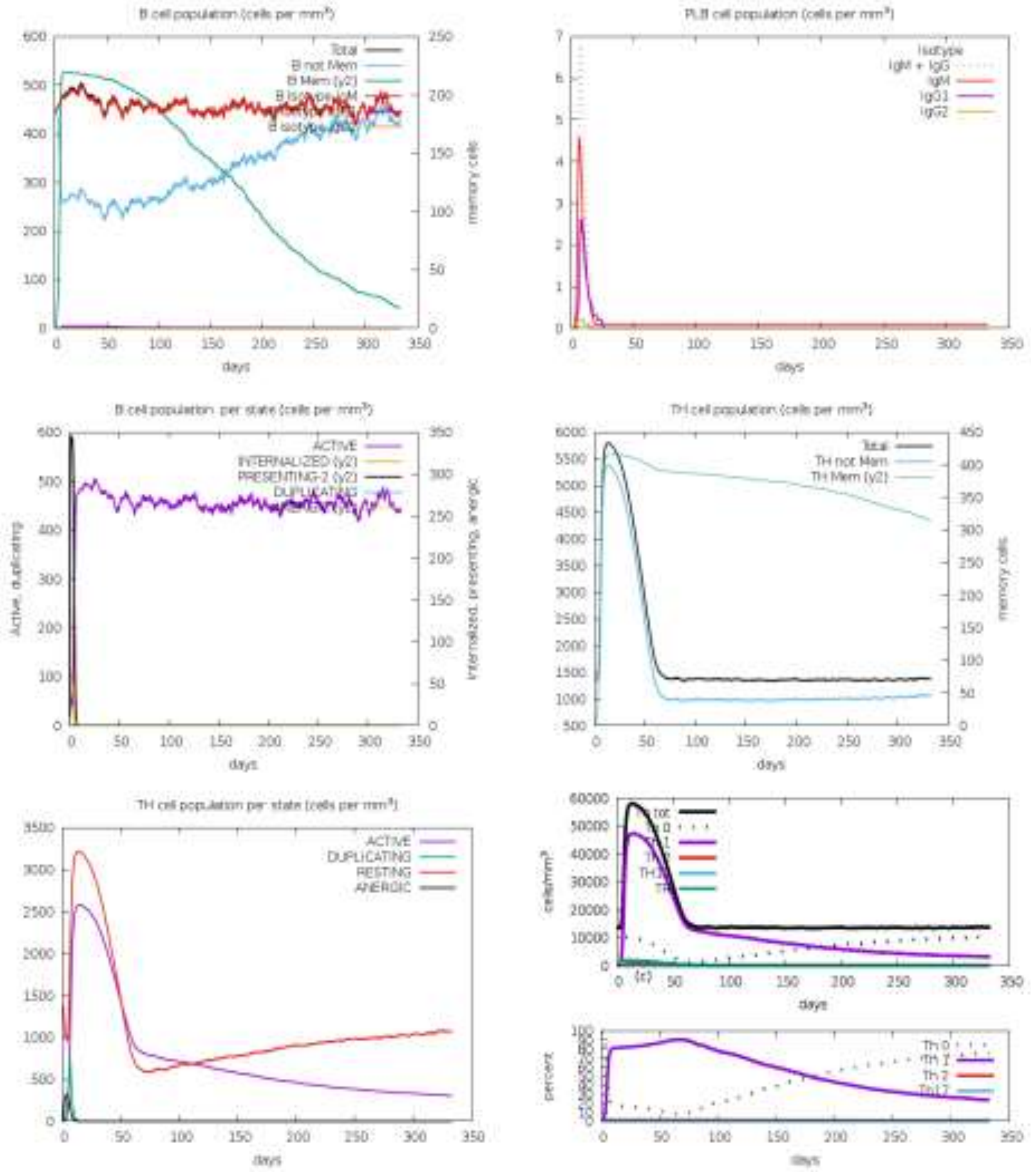

Figure S1. Different B-cell counts in response to the antigen. Legend: Act=active, Intern=internalized the Ag, Pres II = presenting on MHC II, Dup = in the mitotic cycle, Anergic = anergic, Resting = not active.

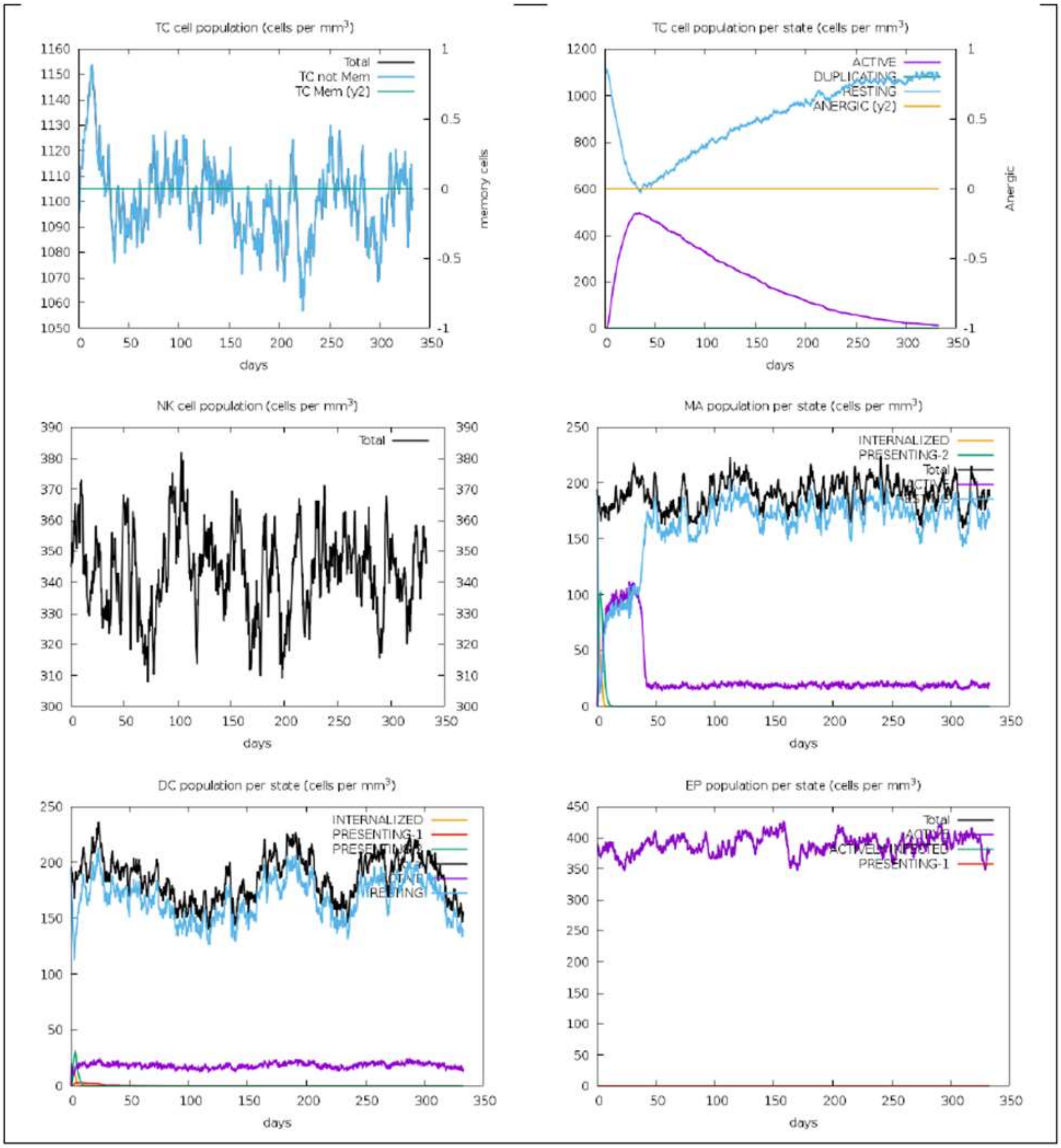

Figure S2. Different T-cell responses to the vaccine; cytotoxic killer T-cells (Tc), natural killer cells (NK), dendritic cells (DC), macrophages (Mφ), and epithelial cells.

Table S1. Predicted B-cell epitopes for shortlisted vaccine targets.

B-CELL EPITOPES	PEPTIDES
>core/451/1/Org1_Gene734 (Lytic transglycosylase domain-containing protein)	IGRYTPADSTQELRDAARRLLPSSSLDKDLERLLRT WHKGYSTKTKRGERPCVNSDTGPYTSDSVYI
	SALPSAVPIQFNPAVKQCIKLYTEERRRLVRY
	GLTINSLVDERLD
	IRMSGGKTDFWEIYPYLPRE
	HIEHEICAGETGVPLATD
	GVSNDELQLLNPQYKRGIIPG
	KKLDKVRDSLYSPDLEASVSDFPEQGGQHSGGSGR TA
	KRANGLKSSSVKPG
	KSSASGTKKKSKYSSKRKSR
>core/1384/1/Org1_Gene274 (superoxide dismutase protein)	FENADLNTI
	FRPGKGGAPK
	AIDKQFGSFEK
	NAGNPVR
	LTYQNRRAD
>core/1029/1/Org1_Gene254 (FKBP-type peptidyl-propyl cis-trans isomerase)	KNADTTAVSEKDSIALSMGILYGQDFANQFEMSRL QGQPIDSVAFLDGFKYGIDTTR
	QLAHDSIDID
	LKDTVSIAMKPADAQAFMQRIQAKKQRENNMKQF GQNIEKGNEYIDTFKKEDGVIVT
	LQEGTGATPS
	KEFDKNEEGIEFA
	YGETGNYTIE

Table S2. Prediction of MHC-I and MHC-II epitopes from B-Cell epitopes.

MHC-I	Percentile score	MHC-II	Percentile score
RTWHKGYSTK	0.02	ERLLRTWHKGYSTK	4.6
ERLLRTWHK	1.4		
RLLPSSSLDK	0.01	AARRLLPSSSLDKDL	7.1
AARRLLPSS	0.91		
KTKRGERPC	0.99	KTKRGERPCVNSDTG	62
RPCVNSDTG	11		
TPADSTQEL	0.04	GRYTPADSTQELRDA	17
DSTQELRDA	11		
GPYTSDSVY	0.04	NSDTGPYTSDSVYI	23
NSDTGPYTS	1.3		
VPIQFNPAV	0.01	AVPIQFNPAVKQCIK	1.2
NPAVKQCIK	4.9		
EERRRLVRY	0.09	IKLYTEERRRLVRY	5.1
IKLYTEERRR	3.2		
LPSAVPIQF	0.01	SALPSAVPIQFNPA	9.8
SAVPIQFNPA	1.3		
TINSLVDER	0.14	GLTINSLVDERLD	2.4
NSLVDERLD	25		
KTDFWEIYPY	0.05	GKTDFWEIYPYLPR	0.15
FWEIYPYLPR	1.3		
MSGGKTDFW	0.05	IRMSGGKTDFWEIYP	18
KTDFWEIYP	2.5		
EICAGETGV	0.6	EICAGETGVPLATD	3.4
ETGVPLATD	12		
EICAGETGV	0.6		
HIEHEICAGE	15	HIEHEICAGETGVP	16
QLLNPQYKR	0.07		
LLNPQYKRGI	3.2	DELQLLNPQYKRGI	1.2
DELQLLNPQY	0.12		
DELQLLNPQY	0.12	GVSNDELQLLNPQYK	1.6
GVSNDELQL	1.1		
DLEASVSDF	1.7	SPDLEASVSDFPEQ	1.3
ASVSDFPEQ	8.9		
KLDKVRDSLY	0.06		

<table>
<tr><td>KVRDSLYSPD</td><td>6.5</td><td>KKLDKVRDSLYSPD</td><td>27.5</td></tr>
<tr><td>GQHSGGSGR</td><td>1.2</td><td rowspan="2">EQGGQHSGGSGRTA</td><td rowspan="2">17</td></tr>
<tr><td>EQGGQHSGG</td><td>22</td></tr>
<tr><td>RANGLKSSS</td><td>2.1</td><td rowspan="2">KRANGLKSSSVKP</td><td rowspan="2">16</td></tr>
<tr><td>GLKSSSVKP</td><td>2.7</td></tr>
<tr><td>KSKYSSKRK</td><td>0.02</td><td rowspan="2">GTKKKSKYSSKRK</td><td rowspan="2">5</td></tr>
<tr><td>GTKKKSKYS</td><td>0.85</td></tr>
<tr><td>SSASGTKKK</td><td>0.09</td><td rowspan="2">KSSASGTKKKSKY</td><td rowspan="2">38</td></tr>
<tr><td>SGTKKKSKY</td><td>0.63</td></tr>
<tr><td>AIDKQFGSF</td><td>0.77</td><td rowspan="2">AIDKQFGSFEK</td><td rowspan="2">9.5</td></tr>
<tr><td>DKQFGSFEK</td><td>6.3</td></tr>
</table>

Table S3. MHC alleles used for epitopes prediction.

MHC class	MHC alleles
MHC-I	HLA-A*01:01, HLA-A*01:01, HLA-A*02:01, HLA-A*02:01, HLA-A*02:03, LA-A*02:03, HLA-A*02:06, HLA-A*02:06, HLA-A*03:01, HLA-A*03:01, HLA-A*11:01, HLA-A*11:01, HLA-A*23:01, HLA-A*23:01, HLA-A*24:02, HLA-A*24:02, HLA-A*26:01, HLA-A*26:01, HLA-A*30:01, HLA-A*30:01, HLA-A*30:02, HLA-A*30:02, HLA-A*31:01, HLA-A*31:01, HLA-A*32:01, HLA-A*32:01, HLA-A*33:01, HLA-A*33:01, HLA-A*68:01, HLA-A*68:01, HLA-A*68:02, HLA-A*68:02, HLA-B*07:02, HLA-B*07:02, HLA-B*08:01, HLA-B*08:01, HLA-B*15:01, HLA-B*15:01, HLA-B*35:01, HLA-B*35:01, HLA-B*40:01, HLA-B*40:01, HLA-B*44:02, HLA-B*44:02, HLA-B*44:03, HLA-B*44:03, HLA-B*51:01, HLA-B*51:01, HLA-B*53:01, HLA-B*53:01, HLA-B*57:01, HLA-B*57:01, HLA-B*58:01, HLA-B*58:01
MHC-II	HLA-DRB1*01:01, HLA-DRB1*03: *04:01, HLA-DRB101,HLA DRB1*04:05,HLADRB1*07:01,HLA,DQA1*03:01/DQB1*03:02,HLADQA1*03:01/DQB1*03:02,HLADQA1*01:02/DQB1*06:02,HLADPA1*02:01/DPB1*01:01,HLADPA1*01:03/DPB1*04:01,HLADPA1*03:01/DPB1*04:02,HLADPA1*02:01/DPB1*05:01,HLADPA1*02:01/DPB1*14:01.

Table S4. Top 20 docked solutions of vaccine for MHC-I.

Solution No	Score	Area	ACE
1	22100	3365.60	492.88
2	20818	2820.00	452.38
3	19576	3631.70	90.02
4	19480	4355.10	166.44
5	19122	2969.20	257.95

6	19014	2841.60	469.81
7	18986	4412.90	129.65
8	18426	4331.90	364.25
9	18352	3367.30	-96.78
10	18346	3688.60	140.99
11	18224	4621.70	-82.44
12	18140	2907.40	342.91
13	17802	3871.20	466.29
14	17798	3487.50	454.04
15	17786	3696.70	452.08
16	17780	3133.00	384.20
17	17754	2747.40	339.24
18	17742	2400.80	454.32
19	17698	3272.10	78.10
20	17694	2845.30	237.79

Table S5. Top 20 docked solutions of vaccine for MHC-II.

Solution No	Score	Area	ACE
1	20106	2672.70	156.38
2	19348	3410.90	-104.98
3	18640	3099.50	242.12
4	18600	2883.70	201.18
5	18332	2629.20	199.37
6	18330	2639.00	296.24

7	17936	2864.50	331.56
8	17574	2230.10	-28.95
9	17458	2394.40	94.76
10	17436	2553.30	476.42
11	17422	2628.90	219.73
12	17354	2717.80	-89.21
13	17076	2601.00	206.56
14	17002	2660.40	97.74
15	16870	2445.50	409.41
16	16844	2865.10	-103.42
17	16684	2206.00	498.34
18	16518	3314.70	57.02
19	16482	2224.30	-34.29
20	16454	2554.80	108.08

Table S6. Top 20 docked solutions of vaccine for TLR-4.

**Solution No**	**Score**	**Area**	**ACE**
1	20450	3270.70	298.46
2	20110	4387.00	496.92
3	19500	2539.30	418.97
4	19482	2689.80	347.35
5	19412	3707.10	28.30

6	19282	2802.80	123.49
7	19230	3481.30	-24.85
8	19122	3374.10	451.65
9	19052	3650.40	-63.35
10	18680	2473.20	-44.07
11	18670	2805.30	315.13
12	18668	2788.40	20.75
13	18488	3260.50	395.06
14	18422	3248.50	297.29
15	18288	3141.90	453.78
16	18084	2685.50	249.18
17	17914	3004.40	199.07
18	17834	3064.90	417.93
19	17820	4090.10	270.23
20	17628	3504.90	219.68
